# Supplementary material for: Predicting visual function by interpreting a neuronal wiring diagram
Source: Nature. 2024 Oct 2;634(8032):113–23. doi: 10.1038/s41586-024-07953-5 (PMC11446822; doi:10.1038/s41586-024-07953-5)
Supplement: Supplementary file 6 — CRF and ERF predictions for individual TmY4 and TmY9 cells. Analogous to Supplementary Data 3, but for TmY target types. Shown are the top four monosynaptic pathways, the strongest pathway passing through each of the top ten intermediary types (ranking from Extended Data Fig. 7), and the trisynaptic pathway Tm1–TmY–Dm3–TmY (see the section entitled Prediction of spatial normalization). [file 41586_2024_7953_MOESM6_ESM.zip › DataS4/TmY9q/720575940622689825.pdf]

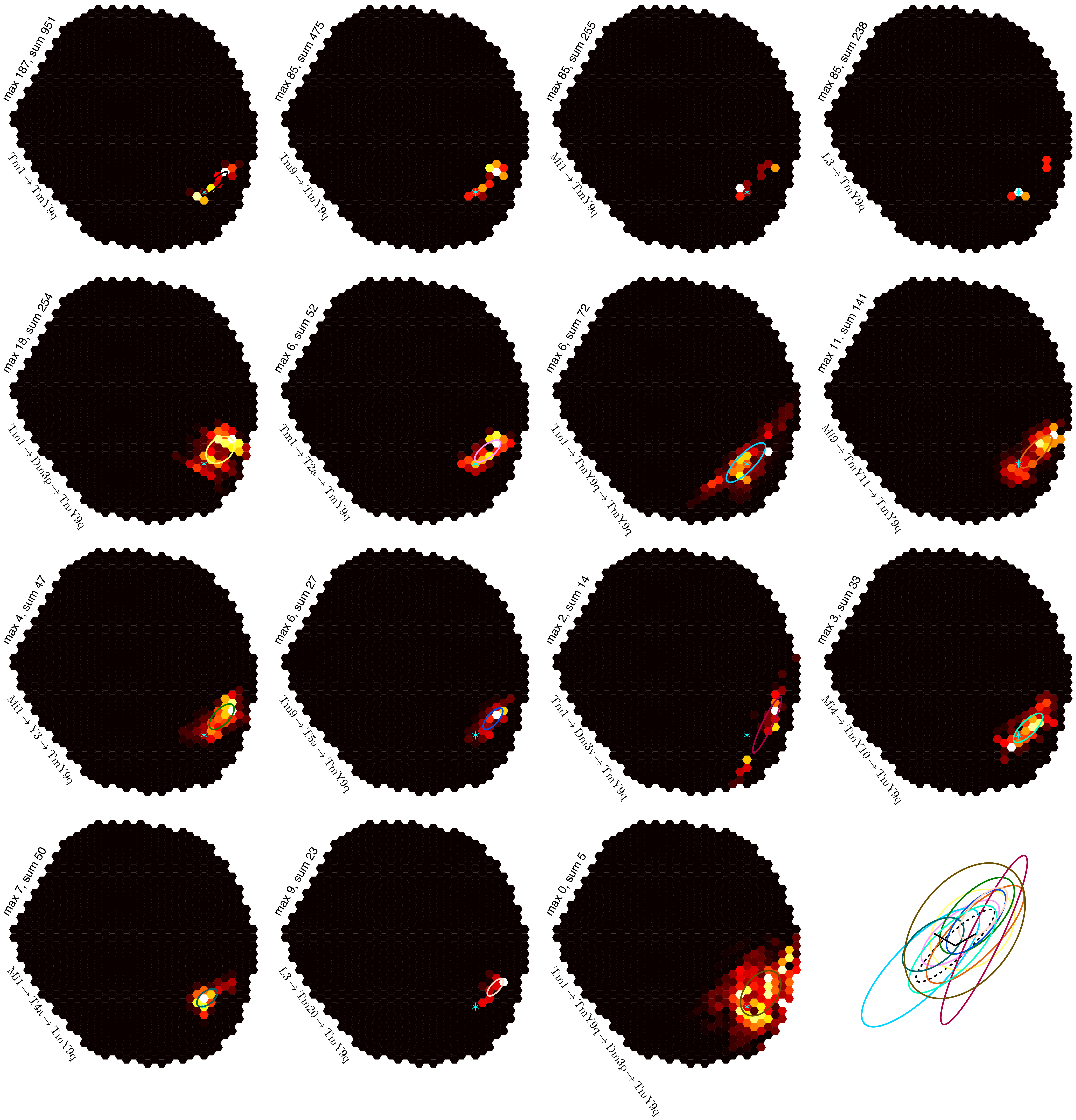

max 187, sum 951
Tm1 → TmY9q
max 85, sum 475
Tm9 → TmY9q
max 85, sum 255
Mi1 → TmY9q
max 85, sum 238
L3 → TmY9q
max 18, sum 254
Tm1 → Dm3p → TmY9q
max 6, sum 52
Tm1 → T2a → TmY9q
max 6, sum 72
Tm1 → TmY9q → TmY9q
max 11, sum 141
Mi9 → TmY11 → TmY9q
max 4, sum 47
Mi1 → Y3 → TmY9q
max 6, sum 27
Tm9 → T5a → TmY9q
max 2, sum 14
Tm1 → Dm3v → TmY9q
max 3, sum 33
Mi4 → TmY10 → TmY9q
max 7, sum 50
Mi1 → T4a → TmY9q
max 9, sum 23
L3 → Tm20 → TmY9q
max 0, sum 5
Tm1 → TmY9q → Dm3p → TmY9q
